# Women Want Choices: Opinions from the Share.Learn.Shape Global Internet Survey About Multipurpose Prevention Technology (MPT) Products in Development

**DOI:** 10.1007/s10461-022-03951-8

**Published:** 2023-03-07

**Authors:** B. A. Friedland, M. Plagianos, C. Savel, V. Kallianes, C. Martinez, L. Begg, K. M. Guthrie, D. Venkatasetty, J. Pickett, L. B. Haddad

**Affiliations:** 1 Center for Biomedical Research, Population Council, 1230 York Avenue, New York, NY 10065 USA; 2 Information Technology, Population Council, New York, NY USA; 3 Borough of Manhattan Community College, New York, NY USA; 4 Department of Psychiatry and Human Behavior, Warren Alpert Medical School of Brown University, Providence, RI USA; 5 Department of Behavioral and Social Sciences, School of Public Health, Brown University, Providence, RI USA; 6 Independent Consultant, Chicago, IL USA

**Keywords:** HIV prevention, Multipurpose prevention technology, End user preferences, STI prevention, cMPT, Contraceptive, Palabras clave, prevención del VIH, tecnología de prevención multipropósito (TPM), preferencias del usuario, prevención de ITS, cMPT, TPM con componente anticonceptivo

## Abstract

Women need multipurpose prevention technologies (MPTs) to simultaneously prevent sexually transmitted infections (STIs), including HIV, with or without contraception. User feedback early in product development is critical for maximizing uptake and continuation. Our global online survey (April 2017–December 2018) explored women’s opinions about MPT formulations in development (e.g., fast-dissolving vaginal inserts, vaginal films, intravaginal rings, injectables, implants), preferences for long-acting or “on-demand” methods, and interest in a contraceptive MPT versus products for HIV/STI prevention alone. Of the 630 women in our final analysis (mean 30 years old; range 18–49), 68% were monogamous, 79% completed secondary education, 58% had ≥ 1 child, 56% were from sub-Saharan Africa and 82% preferred a cMPT versus HIV/STI prevention alone. There were no clear preferences for any specific product or product type (long-acting, on-demand, daily). No single product will appeal everyone, however, adding contraception is likely to increase uptake of HIV/STI prevention methods for most women.

## Resumen

Las mujeres necesitan tecnologías de prevención multipropósito (TPM) para prevenir simultáneamente las infecciones de transmisión sexual (ITS), incluido el VIH, con o sin anticoncepción. Las opiniones de los usuarios cuando un producto comienza a desarrollarse son fundamentales para maximizar la adopción y continuación de dicho producto. Nuestra encuesta global realizada en internet (abril de 2017–diciembre de 2018) exploró las opiniones de las mujeres sobre diferentes fórmulas o dispositivos de TPM que se están desarrollando (ej., insertos vaginales de disolución rápida, láminas vaginales, anillos intravaginales, inyectables, implantes). En esta encuesta se indagó acerca de las preferencias en términos de período de acción (prolongado o breve) y propósito del uso (anticonceptivo, productos para la prevención del VIH/ITS, o ambos). De las 630 mujeres (media de 30 años; rango 18–49) en el análisis final, el 68% eran monógamas, el 79% completaron la educación secundaria, el 58% tenían ≥ 1 hijo, el 56% eran del África subsahariana y el 82% preferían una TPM con componente anticonceptivo en vez de un producto para la prevención de VIH/ITS exclusivamente. No hubo preferencias claras por ningún producto o tipo de producto específico (de acción prolongada, de acción breve, de uso diario). Ningún producto por sí solo logró abarcar todas las preferencias; sin embargo, es probable que la inclusión de métodos anticonceptivos en una TPM aumente el uso de métodos de prevención del VIH/ITS en la mayoría de las mujeres.

## Introduction

Women of reproductive age worldwide face two overlapping risks that impact their health and well-being: unintended pregnancy and sexually transmitted infections (STIs), including HIV. Globally, nearly half of all pregnancies each year are unintended; over 60% of those end in abortion, contributing significantly to maternal morbidity and mortality [1]. Nearly one-quarter of women living in low- and middle-income countries (LMICs) have an unmet need for contraception, a rate that rises to nearly 50% among 15–19-year-olds in the same regions [2]. Furthermore, STI rates have been increasing globally [3], leading to significant morbidity, poor birth outcomes, infertility, cervical cancer, and increased risk of HIV [4, 5]. Despite advances in HIV treatment and prevention over the last decade, AIDS continues to be the leading cause of death globally among women of reproductive age [6]. Yet, growing evidence indicates that many women are more worried about unintended pregnancy and STIs other than HIV and many would be more likely to use an HIV/STI prevention method that is also a contraceptive [7–14]. Currently, condoms are the only “multipurpose prevention technology” (MPT) products that can simultaneously prevent pregnancy and most STIs, including HIV [15, 16]. However, male condoms require women to engage in often difficult negotiations with their partners [15, 17], and female condoms have had limited uptake in most populations due to barriers to access and lack of acceptability, particularly among male partners [18, 19]. As demonstrated by the contraceptive field, a wider range of methods will help women select products that they are less apt to discontinue, increase uptake by first-time users, and ultimately enable more women to protect themselves throughout their lives [20, 21].

According to the Initiative for Multipurpose Prevention Technologies (IMPT) database, there are currently 25 MPTs in different stages of development for prevention of HIV and other STIs, with and without contraception, in a variety of formulations, such as intravaginal rings (IVRs), implants, injectables, vaginal films, fast-dissolving vaginal inserts (FDIs) or tablets, douches, and microarray patches (MAPs) [22]. In addition to the rigorous safety and efficacy requirements for regulatory approval of new products, end-user research conducted as early as possible during product development is important to ensure that methods meet users’ needs [23–31]. A number of recent studies explored women’s opinions about product delivery platforms using placebo products [12, 23, 26, 29–31]. Placebo studies allow women to formulate their opinions based on actual experience using proxy products, however, such studies are limited in demographic and geographic scope and, in some cases, number of participants. Conducting surveys using the internet enables enrollment of a broader range of participants than would be feasible through in-person methods at relatively low cost. Internet-based surveys have been gaining momentum in recent years in the context of HIV-prevention [9, 32–34].

In this paper, we report on Share.Learn.Shape—the first cross-sectional, global internet survey to explore women’s interest in using nine different HIV/STI prevention methods in development, and whether they prefer a contraceptive MPT (cMPT) versus an HIV/STI prevention method only.

## Methods

### Survey Design

We developed Share.Learn.Shape as an interactive questionnaire system (iQS) survey that could be taken on any device with web access including mobile phones, laptops, tablets or desktop computers. The survey was available in English and Spanish and included illustrations of existing contraceptive methods [such as injectables, oral contraceptives, intrauterine devices (IUDs), and condoms] and animated video clips of four novel vaginal products in development (IVR, gel, FDI, and film) for women to see how these products would be used. We collected information on background demographics, experiences using HIV/STI/pregnancy prevention methods, and elicited opinions about nine specific products in development: five designed for “on-demand” use (vaginal gel used before or after sex, vaginal film, FDI, or oral pill used before sex); three longer acting methods (IVR, injectable or implant); or a daily oral pill. Most questions (15/17) were multiple choice with radio buttons or drop-down menus; two questions were free text fields in which women had to type in responses about “definite musts” and “definite no’s” for any HIV/STI prevention product they would want to use. Once a participant started the survey, she had up to 30 min to complete it. Although the survey was expected to take only 15–20 min, the consent form advised women that if they did not have one half hour available, they should wait to initiate the survey until they had enough time to finish it.

### Survey Programming and Security

The Share.Learn.Shape survey was entirely web-based and used free and open-source (FOSS) technology for programming. The survey website was hosted on an Apache 2.4 server (Dreamhost; Brea, CA, USA) running the Ubuntu Linux operating system (Canonical LTD). We programmed the survey using Personal Home Page (PHP; The PHP Group) and responses were saved in a MySQL database. The survey website was secured using standard protocols so that all data to and from the server were encrypted. Access to the database was limited to the Population Council’s Information Technology (IT) personnel via a username and password. Only Population Council IT staff could download the survey data, which was then shared with Population Council data management in a .csv format for analysis. At the end of data collection, the survey and database were backed up and archived by the Population Council. The survey was then taken offline and was no longer available for further data collection.

To preserve respondents’ anonymity, two data tables were created: one table recording informed consent and a second table recording the survey responses. No personally identifiable information was collected. When a respondent accessed the survey website, an anonymous encrypted token was created, and a new record was added to the consent table. If the respondent consented, the anonymous token was added to the survey response table, and she was able to begin the survey. The unique token, survey date, and time that was stored in the database for each participant who initiated the survey ensured that only responses from participants who had consented were recorded. No one could take the survey without first giving consent and the only connection between the consent table and the survey response table was the token.

The survey was initially programmed so that respondents had to view the video clips of products in development before answering questions about their interest in using them. However, based on results of pilot testing, we noted that many respondents stopped the survey at the point that the video clips started. We hypothesized that the majority of those who ended early had low bandwidth and could not view the videos. Therefore, we reprogrammed the final survey so that respondents could answer all questions with or without watching the video clips and added an illustration of the four products in case people could not or chose not to view the videos. To reduce potential bias related to product order, the questions about interest in the nine products appeared in a random order; each of the nine products was equally likely to appear in any given order within this set of questions.

After completing the survey, the survey window closed automatically, and respondents were redirected to another web page thanking them for participating. The thank you page also contained a “badge” acknowledging that the participant had contributed to HIV-prevention efforts, with links to share the badge with their friends to encourage them to take the survey.

### Study Population and Recruitment Strategies

Cisgender females aged 18–49 years old who had sex with a male at least once in their lifetime were eligible. We engaged a digital marketing, design, and branding agency to create an appealing name and design for the survey landing page to attract potential respondents. We chose “Share.Learn.Shape” to engage women without mentioning HIV/STIs overtly, and the artwork included photographs of women of different ages, races, and ethnicities to encourage broad participation.

We recruited a convenience sample of participants via list-serves, news groups, social media, and other relevant platforms. Where feasible, researchers conducting studies in South Africa and Zimbabwe encouraged their participants to take the survey while they waited for study-related appointments. Respondents were not emailed directly by the research team; however, it was possible that women might receive emails from people within their own networks who may have heard about the survey. Any woman finding out about the survey via any avenue was able to click on the link to the survey.

### Data Management and Statistical Analysis

#### Data Management

Survey data were maintained in SAS data sets from the downloaded .csv files, which were stored on secure network drives with restricted read/write access. Data management reviewed data completeness and integrity prior to analysis. Given the self-collected, anonymous nature of the survey, querying of incomplete data was not possible. However, data checks were programmed to alleviate potential for illogical responses (such as responding that a specific contraceptive method had been used while also responding that no contraceptive methods had ever been used).

#### Sample Size Calculation

We planned to collect as many responses as possible during the data collection period, aiming to have balance among five regions (Asia, Europe, Latin America, sub-Saharan Africa, and North America). We had determined a priori that a sample of at least 200 women would enable us to detect if respondents thought they would be more likely to use a cMPT versus an HIV/STI prevention method alone by at least 10% with ≥ 80% power.

#### Data Analysis

We summarized demographic and other baseline characteristics related to pregnancy and HIV/STI prevention methods and vaginal product use among women who began the study (“Eligible”) and those in the “Product Interest Subgroup,” defined as the subset of women who responded to at least one question about the nine hypothetical prevention products. We categorized the nine hypothetical products into three different product types: on-demand (products used before or after sex, such as gels, FDIs, and oral pills), long-acting (products designed for continuous use for at least 1 month, whether provider- or user-administered), including IVRs, injectables and implants), and a daily pill. We then summarized interest in a cMPT and the three product types by the following characteristics: demographics (age, marital status, education, parity, geographic region), contraceptive and HIV/STI prevention methods ever used, previous vaginal product use, current prevention needs, and formulation preference. Additionally, we compared interest in a cMPT by interest in “on-demand” products, long-acting products, and a daily pill. Respondents who were “somewhat” or “very” interested in using each method were included in the “interested” group. To identify characteristics that were predictive of interest in each of the four different product types, we fit bivariate logistic regression models for each product type and baseline characteristic, calculating the odds ratios (ORs) of interest. We then fit multivariate logistic regression models for each product type and for a cMPT. All variables mentioned above were initially considered in the models; we then repeated a backward elimination process, removing characteristics that were not significant (Wald chi-square, p ≥ 0.05), although we retained age in all models, regardless of significance.

### Ethics

The protocol and informed consent form were reviewed and approved by the Population Council Institutional Review Board (New York, NY, USA). Through the online informed consent process, respondents were told their participation would be anonymous and their responses would remain confidential. Respondents provided electronic informed consent by clicking “I agree” (versus “I do not agree”) at the bottom of the informed consent web page before being redirected to the survey. Respondents did not receive compensation for participation.

## Results

### Background Characteristics

Between April 2017 and December 2018, 852 women consented to take the survey, 737 met eligibility criteria, and 630 answered at least one question about product preferences—the Product Interest Subgroup (PIS)—and were included in the final analysis. More than half of respondents were from sub-Saharan Africa and approximately two-thirds were monogamous (Table 1). There were several differences between women from high-income countries (HICs) versus low-and-middle-income countries (LMICs). For example, participants were 30 years old on average, overall (range 18–49); however, 28% of respondents from LMICs were in the 18–24-year-old age group, compared to 13% of respondents from HICs (chi-square = 20.2, p = 0.0002; data not shown). Overall, 75% of respondents had completed secondary education, however, 91% from HICs had more than secondary education compared to 55% from LMICs (chi-square = 109.2, p < 0.0001; data not shown). More than half of respondents had at least one child, 65% of whom were from LMICs versus 30% from HICs (chi-square = 69.6, p < 0.0001; data not shown).

**Table 1:** Demographics and background characteristics (n = 737)

	Eligible (N = 737)	Product interest subgroup (N = 630)
Age, mean (range)	30.4, 18.0–48.9	30.4, 18.0–48.9
18–24	182 (25%)	164 (26%)
25–34	367 (50%)	303 (48%)
35–44	157 (21%)	137 (22%)
45+	31 (4%)	28 (4%)
Marital status		
Has husband/steady partner	477 (65%)	428 (68%)
Has husband/steady partner plus other male sexual partners	49 (7%)	45 (7%)
Has multiple sexual partners, but no steady partner	35 (5%)	31 (5%)
No current partner, but ≥ 1 male sexual partner in lifetime	102 (14%)	88 (14%)
Female partner(s)	4 (1%)	3 (< 1%)
Not answered^a^	70 (9%)	33 (5%)
Years of education		
No formal schooling	44 (6%)	16 (3%)
1–8 years	73 (10%)	59 (9%)
Some high school/secondary	64 (9%)	55 (9%)
12 (completed secondary)	150 (20%)	135 (21%)
More than 12 years (tertiary)	406 (55%)	365 (58%)
Number of children		
None	303 (41%)	265 (42%)
1	194 (26%)	173 (27%)
2	145 (20%)	132 (21%)
3 or more	74 (10%)	56 (9%)
Primary residence		
South Africa	292 (40%)	267 (42%)
Other Sub-Saharan Africa	109 (15%)	88 (14%)
USA	136 (18%)	120 (19%)
Latin America	84 (11%)	68 (11%)
Europe/Canada/Australia	44 (6%)	38 (6%)
Other^b^	14 (2%)	11 (2%)
Not answered^a^	58 (8%)	38 (6%)
Family planning strategies ever used^c^		
Male condoms	463 (63%)	419 (67%)
Pill	364 (49%)	330 (52%)
Injectable	223 (30%)	204 (32%)
Withdrawal	211 (29%)	184 (29%)
IUD	174 (24%)	157 (25%)
Implant	108 (15%)	100 (16%)
Female condoms	88 (12%)	79 (13%)
Timing/safe days	86 (12%)	81 (13%)
Intravaginal ring	50 (7%)	44 (7%)
Sterilization (female)	24 (3%)	22 (3%)
Sterilization (male)	22 (3%)	20 (3%)
Vaginal spermicide	31 (4%)	29 (5%)
Diaphragm	22 (3%)	21 (3%)
Other^d^	22 (3%)	21 (3%)
Never used FP	25 (3%)	21 (3%)
Not answered^a^	53 (7%)	13 (3%)
HIV/STI prevention strategies ever used^c^		
Male condoms	575 (78%)	520 (83%)
One partner, exclusive	241 (33%)	212 (34%)
No anal sex	121 (16%)	108 (17%)
Female condoms	98 (13%)	90 (14%)
Limit number of partners	96 (13%)	85 (13%)
Partner been tested, no STDs	61 (8%)	55 (9%)
No oral sex	31 (4%)	24 (4%)
Use PEP	11 (1%)	10 (2%)
Use PREP	0 (0%)	0 (0%)
No vaginal sex	4 (1%)	4 (1%)
Have never used anything to prevent HIV/STDs	15 (2%)	14 (2%)
Not answered^a^	66 (9%)	21 (3%)
Vaginal products ever used^c^		
Tampons with applicator	281 (38%)	261 (41%)
Tampons without applicator	284 (39%)	256 (41%)
Vaginal medication	259 (35%)	240 (38%)
Commercial sexual lubricant	245 (33%)	234 (37%)
Water	238 (32%)	209 (33%)
Other sexual lubricant (lotion, saliva, oil, etc.)	150 (20%)	144 (23%)
Douche	61 (8%)	54 (9%)
Prefer not to answer	22 (3%)	20 (3%)

^a^Includes not answered and prefer not to answer

^b^Other countries include Australia (3), Azerbaijan (1), Canada (4), Egypt (3), India (3), Mongolia (1), Nepal (1), Philippines (1), United Arab Emirates (1)

^c^Responses do not add up to 100% as participants could choose more than one option

^d^Other contraceptive includes patch, emergency contraception

### Previous Family Planning, HIV/STI Prevention and Vaginal Product Use

Male condoms (63%), oral contraceptives (49%) and injectables (30%) were the most common family planning methods ever used and only 3% of participants reported never using any method (Table 1). The two most common strategies ever used for HIV/STI prevention were male condoms (78%) and mutual monogamy (33%); only 2% of respondents had never used any of the listed strategies to prevent HIV/STIs. Vaginal product use was relatively common, with approximately one-third having used tampons (with or without applicators), vaginal medication, or commercial lubricants.

### Product Interest

**Fig. 1:**
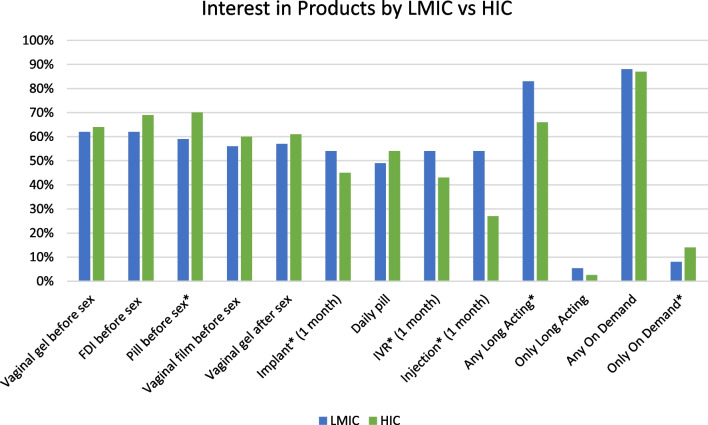
Interest in MPT products, by low- and middle-income (LMIC) vs high-income countries (HIC), product interest sub-group (PIS) (n = 630). *Indicates significant (chi-square p < 0.05) difference between LMIC and HIC responses: pill before sex chi-square = 5.32 p = 0.021; implant chi-square = 4.49 p = 0.034; IVR chi-square = 5.49 p = 0.019; injection chi-square = 34.2 p < 0.0001; any long acting chi-square = 19.4 p < 0.0001; only on demand chi-square = 4.15 p = 0.042. *Long acting* includes implant, IVR, injection; *On demand* includes vaginal gel before sex, FDI before sex, pill before sex, vaginal film before sex, vaginal gel after sex

As shown in Fig. 1, at least half of the PIS respondents were interested in any one of the nine products they were asked about, with no clear preferences among the products. Most women were interested in using both on-demand and long-acting methods, with only a small proportion of women wanting only on-demand methods or only long-acting methods. Some differences in preferences emerged when comparing women from LMICs to those from HICs; more women from LMICs than from HICs were interested in long-acting methods: implant (56% vs 46%, chi-square = 4.49 p = 0.034), IVR (57% vs 46%, chi-square = 4.49 p = 0.034), injection (73% vs 46% chi-square = 34.2 p < 0.0001) and any long acting (83% vs 66% chi-square = 19.4 p < 0.0001). More women from HICs than LMICs were interested in using a pill before sex (41% vs 30%, chi-square = 5.32 p = 0.021), or only an on-demand method (14% vs 8% chi-square = 4.15 p = 0.042).

More than three-quarters of the respondents were interested in using a product to prevent HIV and almost two-thirds were interested in using products for pregnancy or STI prevention. The majority of women (82%; 95% CI 79–85%) said that if an HIV or other STI prevention product could also prevent unintended pregnancy, they would be more likely to use it (Table 2). 30% of respondents were interested in using a product that could be used vaginally and 4% were interested in a product that could be used rectally. 6% of participants said they were not interested in any of the product types listed. Those interested in vaginally applied on-demand methods (n = 531) had varied preferences for applying the products with an applicator (31%), with their fingers without an applicator (25%), or using either fingers or an applicator (31%). Most preferred products that would dissolve within a few seconds (65%); increase (38%) or have no impact on vaginal lubrication (35%); and would feel warm (28%) or have no noticeable impact on vaginal temperature (36%) during or after dissolution.

**Table 2:** Women’s interest in product characteristics, product interest subgroup (n = 630)

Interest in product types^a^	n (%)
Product to prevent HIV	481 (76%)
Product to prevent STDs (like herpes, HPV, gonorrhea, chlamydia, syphilis)	392 (62%)
Product to prevent pregnancy	403 (64%)
HIV/STI prevention product that also prevents unintended pregnancy	**518 (82%)**
Product that could be used vaginally (inside the vagina)	186 (30%)
Product that could be used rectally (inside the butt)	27 (4%)
Not interested in any of these	35 (6%)
Prefer not to answer	5 (1%)
Characteristics of on-demand^b^ (gel, film, or vaginal tablet) products	n = 531
Application preference	
Inserted with your finger only, without an applicator	134 (25%)
Inserted with an applicator	166 (31%)
Either method would be OK	163 (31%)
Neither of these appeals to me	14 (3%)
I am not sure	17 (3%)
Not answered^c^	32 (6%)
Lubrication preference	
Adds lubrication or makes your vagina feel wetter as it dissolves	201 (38%)
Reduces lubrication or makes your vagina feel dryer as it dissolves	30 (6%)
Has no effect on lubrication or vaginal wetness	186 (35%)
Any of the above would be ok with me	36 (7%)
I am not sure	47 (9%)
Not answered^c^	31 (6%)
Temperature preference	
Feels warm as it dissolves	149 (28%)
Feels cool as it dissolves	60 (11%)
Does not feel warm or cool as it dissolves	192 (36%)
Any of the above would be ok with me	70 (13%)
I am not sure	38 (7%)
Not answered^c^	22 (4%)
Dissolution preference	
Dissolves within a few seconds	347 (65%)
Dissolves within 10 min	69 (13%)
Dissolves within one hour	15 (3%)
Any of the above would be ok with me	44 (8%)
I am not sure	28 (5%)
Not answered^c^	28 (5%)

Bold value indicates significant findings

^a^Does not add up to 100% as more than one response possible

^b^Denominator is women who said they were interested in gel, film, or vaginal tablet products (n = 531)

^c^Not answered incudes missing and prefer not to answer

As shown in Table 3, among those who listed “must have’s” or “definite no’s” for any HIV/STI prevention product in the free text field (n = 450), the most commonly cited requirements were safety (45%) and affordability (24%), with many also citing efficacy (16%), ease of use (15%), comfort (15%), and impact on sex (12%). Women had a wide range of opinions about specific product formulations that would meet these criteria. For example, some women only wanted long-acting, highly efficacious products whereas others wanted only products that were non-systemic that could be used at the time of sex.

**Table 3:** Most important characteristics of future HIV/STI prevention products—responses to free text fields regarding “must haves” and “definite nos” (n = 450)

Characteristic	n (%)
Safe	202 (45%)
Affordable	108 (24%)
Effective	71 (16%)
Easy to use	67 (15%)
Comfortable	66 (15%)
Doesn’t interfere with sex	52 (12%)
Contraceptive	41 (9%)
Accessible	40 (9%)
Adds lubrication	33 (7%)
Non-hormonal	24 (5%)
Daily dosing	24 (5%)
Discrete	23 (5%)
Available over the counter	21 (5%)
Natural/neutral/pleasant smell	18 (4%)
Non-vaginal	16 (4%)
Messy	14 (3%)
Non-injectable	13 (3%)
Interferes with fertility	10 (2%)

### Predictors of Interest in Product Types

As shown in Tables 4, 5, 6, and 7, we fit models to identify predictors of interest in cMPTs, on-demand or long-acting methods, or a daily pill.

**Table 4:** Predictors of preference for cMPT, product interest sub-group (n = 630)

Characteristic	Interested n = 518	Not interested n = 112	Unadjusted odds ratio (95% CI)	Adjusted odds ratio (95% CI)
Age				
Age 18–24	**134 (26%)**	**28 (25%)**	**1.05 (0.65–1.68)**	**1.12 (0.69–1.83)**
Age 25+	384 (74%)	84 (75%)	Ref	
Prevention needs^a^				
Interest STI prevention	344 (66%)	48 (43%)	2.64 (1.74–4.00)	
HIV prevention	**411 (79%)**	**70 (63%)**	**2.31 (1.49–3.57)**	**1.70 (1.06–2.70)**
Pregnancy	**351 (68%)**	**52 (46%)**	**2.43 (1.60–3.67)**	**1.81 (1.16–2.82)**
Formulation preference^a^				
Product for vaginal use	**170 (33%)**	**16 (14%)**	**2.93 (1.67–5.13)**	**2.15 (1.19–3.89)**
Product for rectal use	24 (5%)	3 (3%)	1.77 (0.52–5.98)	

Bold values indicate significant findings

Marital status, education, parity, primary residence, contraceptive methods ever used, HIV/STI prevention strategies ever used and prior vaginal product use were not associated with preference for a cMPT (Wald chi-square p > 0.05)

^a^May add up to more than 100% as more than one response was possible

**Table 5:** Predictors of interest in using on-demand products, product interest sub-group (n = 630)

Characteristic	Interested n = 551	Not interested n = 79	Unadjusted odds ratio (95% CI)	Adjusted odds ratio (95% CI)
Age				
Age 18–24	**145 (26%)**	**17 (22%)**	**1.30 (0.74–2.30)**	**1.35 (0.73–2.50)**
Age 25+	406 (74%)	62 (78%)	Ref	
Marital status				
Has husband/steady partner	369 (67%)	59 (75%)	Ref	
Has multiple sexual partners with or without steady partner	72 (13%)	4 (5%)	2.88 (1.01–8.12)	
No current partner	76 (14%)	12 (15%)	1.01 (0.52–1.98)	
Other/not answered^a^	34 (6%)	4 (5%)	1.36 (0.47–3.97)	
Primary residence				
Sub-Saharan Africa	309 (56%)	46 (58%)	Ref	
US/Europe/Canada/Australia	**138 (25%)**	**20 (25%)**	**1.03 (0.59–1.80)**	**1.69 (0.81–3.52)**
Latin America	**65 (12%)**	**3 (4%)**	**3.23 (0.97–10.69)**	**4.08 (1.17–14.19)**
Other^b^	7 (1%)	4 (5%)	0.26 (0.07–0.93)	0.21 (0.04–1.12)
Not answered^a^	32 (6%)	6 (8%)	0.79 (0.32–2.00)	1.02 (0.39–2.67)
Contraceptive methods ever used^b^				
Male condoms	365 (66%)	54 (68%)	0.91 (0.55–1.51)	
Female condoms	**78 (14%)**	**1 (1%)**	**12.86 (1.76–93.80)**	**12.56 (1.65–95.64)**
Oral contraceptive pill	288 (52%)	42 (53%)	0.97 (0.60–1.55)	
Injectable	**185 (34%)**	**19 (24%)**	**1.60 (0.93–2.75)**	**1.90 (1.04–3.50)**
Implant	84 (15%)	16 (20%)	0.71 (0.39–1.29)	
IUD	132 (24%)	25 (32%)	0.68 (0.41–1.14)	
Intravaginal ring	**35 (6%)**	**9 (11%)**	**0.53 (0.24–1.14)**	**0.29 (0.11–0.78)**
Sterilized	519 (94%)	78 (99%)	4.81 (0.65–35.69)	
Withdrawal	169 (31%)	15 (19%)	1.89 (1.05–3.41)	
Timing/safe days	73 (13%)	8 (10%)	1.36 (0.63–2.93)	
Spermicide or diaphragm	**51 (9%)**	**2 (3%)**	**3.93 (0.94–16.46)**	**7.74 (1.37–43.72)**
None/never used	16 (3%)	6 (8%)	0.36 (0.14–0.96)	
Methods ever used to prevent HIV and other STIs^b^				
Male condoms	458 (83%)	62 (78%)	1.35 (0.76–2.42)	
Female condoms	87 (16%)	3 (4%)	4.75 (1.47–15.40)	
Prevention needs^c^				
Interest STI prevention	355 (64%)	37 (47%)	2.06 (1.28–3.31)	
HIV prevention	**435 (79%)**	**46 (58%)**	**2.69 (1.65–4.40)**	**2.09 (1.18–3.70)**
Pregnancy	360 (65%)	43 (54%)	1.58 (0.98–2.54)	
Contraceptive MPT				
More likely to use HIV/STI prevention product that includes contraception	**497 (90%)**	**59 (75%)**	**0.32 (0.18–0.57)**	**2.07 (1.18–3.65)**
Formulation preference^c^				
Product for vaginal use	**177 (32%)**	**9 (11%)**	**3.68 (1.80–7.54)**	**2.63 (1.20–5.76)**
Product for rectal use	25 (5%)	2 (3%)	1.83 (0.43–7.88)	

Bold values indicate significant findings

Education, parity and prior vaginal product use were not associated with interest in an on-demand product (Wald chi-square p > 0.05)

^a^Not answered includes missing and prefer not to answer

^b^Other countries include Azerbaijan (1), Egypt (3), India (3), Mongolia (1), Nepal (1), Philippines (1), United Arab Emirates (1)

^c^May add up to more than 100% as more than one response was possible

**Table 6:** Predictors of interest in using long-acting methods, product interest sub-group (n = 630)

Characteristic	Interested in a long-acting product n = 490	Not interested in a long-acting product n = 140	Unadjusted odds ratio (95% CI)	Adjusted odds ratio (95% CI)
Age				
Age 18–24	**134 (27%)**	**28 (20%)**	**1.51 (0.95–2.38)**	**0.99 (0.59–1.64)**
Age 25+	356 (73%)	112 (80%)	Ref	Ref
Years of education				
No formal schooling	12 (2%)	4 (3%)	0.40 (0.12–1.31)	0.52 (0.15–1.85)
1–12 years	220 (45%)	29 (21%)	Ref	Ref
More than 12 years	**258 (53%)**	**107 (76%)**	**0.32 (0.20–0.50)**	**0.46 (0.28–0.77)**
Number of children				
0	199 (41%)	70 (50%)	Ref	
1 or more	291 (59%)	70 (50%)	1.46 (1.00–2.13)	
Primary residence				
Sub-Saharan Africa	302 (62%)	53 (38%)	Ref	
US/Europe/Canada/Australia	104 (21%)	54 (39%)	0.34 (0.22–0.53)	
Latin America	51 (10%)	17 (12%)	0.53 (0.28–0.98)	
Other^a^	6 (1%)	5 (4%)	0.21 (0.06–0.72)	
Not answered^b^	27 (6%)	11 (8%)	0.43 (0.20–0.92)	
Contraceptive methods ever used^c^				
Male condoms	315 (64%)	104 (74%)	0.62 (0.41–0.95)	
Female condoms	68 (14%)	11 (8%)	1.89 (0.97–3.67)	
Oral contraceptive pill	251 (51%)	79 (56%)	0.81 (0.56–1.18)	
Injectable	**182 (37%)**	**22 (16%)**	**3.17 (1.94–5.18)**	**2.18 (1.27–3.74)**
Implant	**90 (18%)**	**10 (7%)**	**2.93 (1.48–5.79)**	**2.43 (1.17–5.02)**
IUD	124 (25%)	33 (24%)	1.10 (0.71–1.71)	
Intravaginal ring	32 (7%)	12 (9%)	0.75 (0.37–1.49)	
Sterilized	25 (5%)	8 (6%)	0.89 (0.39–2.01)	
Withdrawal	138 (28%)	46 (33%)	0.80 (0.54–1.20)	
Timing/Safe days	57 (12%)	24 (17%)	0.64 (0.38–1.07)	
Spermicide or diaphragm	40 (8%)	13 (9%)	0.87 (0.45–1.67)	
None/never used	17 (3%)	5 (4%)	0.97 (0.35–2.68)	
Vaginal product ever used^c^				
Tampons	273 (56%)	87 (62%)	0.77 (0.52–1.13)	
Vaginal medication	184 (38%)	56 (40%)	0.90 (0.61–1.33)	
Lubricant	195 (40%)	69 (49%)	0.68 (0.47–0.99)	
Water/Douche	184 (38%)	48 (34%)	1.15 (0.78–1.71)	
Prevention needs^c^				
Interest STI prevention	314 (64%)	78 (56%)	1.42 (0.97–2.08)	
HIV prevention	**396 (81%)**	**85 (61%)**	**2.73 (1.82–4.10)**	**1.98 (1.27–3.07)**
Pregnancy	318 (65%)	85 (61%)	1.20 (0.81–1.76)	
Contraceptive MPT				
More likely to use HIV/STI prevention product that includes contraception	**425 (87%)**	**93 (66%)**	**3.30 (2.13–5.12)**	**3.14 (1.96–5.04)**
Formulation preference^c^				
Product for vaginal use	156 (32%)	30 (21%)	1.71 (1.10–2.68)	
Product for rectal use	21 (4%)	6 (4%)	1.00 (0.40–2.53)	

Bold values indicate significant findings

Marital status and prior HIV/STI prevention methods were not associated with interest in a long acting method (Wald chi-square p > 0.05)

^a^Other includes United Arab Emirates (n = 1), Azerbaijan (n = 1), Egypt (n = 3), India (n = 1), Mongolia (n = 1), Nepal (n = 1), Philippines (n = 1)

^b^Not answered includes missing and prefer not to answer

^c^May add up to more than 100% as more than one response was possible

**Table 7:** Predictors of interest in using a daily pill, product interest sub-group (n = 630)

Characteristic	Interested n = 320	Not interested n = 310	Unadjusted odds ratio (95% CI)	Adjusted odds ratio (95% CI)
Age				
Age 18–24	**83 (26%)**	**79 (25%)**	**1.02 (0.72–1.46)**	**0.97 (0.67–1.41)**
Age 25+	237 (74%)	231 (75%)	Ref	
Marital status				
Has husband/steady partner	210 (66%)	218 (70%	Ref	
Has multiple sexual partners with or without steady partner	47 (15%)	29 (9%)	1.68 (1.02–2.77)	
No current partner	45 (14%)	43 (14%)	1.09 (0.69–1.72)	
Other/not answered^a^	18 (6%)	20 (6%)	0.93 (0.48–1.82)	
Contraceptive methods ever used^b^				
Male condoms	215 (67%)	204 (66%)	1.06 (0.76–1.48)	
Female condoms	53 (17%)	26 (8%)	2.17 (1.32–3.57)	
Oral contraceptive pill	176 (55%)	154 (50%)	1.24 (0.91–1.69)	
Injectable	113 (35%)	91 (29%)	1.31 (0.94–1.84)	
Implant	47 (15%)	53 (17%)	0.84 (0.55–1.28)	
IUD	**64 (20%)**	**93 (30%)**	**0.58 (0.40–0.84)**	**0.59 (0.41–0.86)**
Intravaginal ring	22 (7%)	22 (7%)	0.97 (0.52–1.78)	
Sterilized	16 (5%)	17 (5%)	0.91 (0.45–1.83)	
Withdrawal	93 (29%)	91 (29%)	0.99 (0.70–1.39)	
Timing/safe days	35 (11%)	46 (15%)	0.71 (0.44–1.13)	
Spermicide or diaphragm	31 (10%)	22 (7%)	1.40 (0.79–2.48)	
None/never used	11 (3%)	11 (4%)	0.97 (0.41–2.27)	
Methods ever used to prevent HIV and other STIs^b^				
Male condoms	269 (84%)	251 (81%)	1.24 (0.82–1.87)	
Female condoms	**62 (19%)**	**28 (9%)**	**2.42 (1.50–3.90)**	**2.15 (1.32–3.50)**
Prevention needs^b^				
Interest STI prevention	213 (67%)	179 (58%)	1.46 (1.05–2.01)	
HIV prevention	**267 (83%)**	**214 (69%)**	**2.26 (1.55–3.31)**	**1.88 (1.26–2.80)**
Pregnancy	216 (68%)	187 (60%)	1.37 (0.99–1.89)	
Contraceptive MPT				
More likely to use HIV/STI prevention product that includes contraception	274 (86%)	244 (79%)	1.61 (1.07–2.44)	
Formulation preference^b^				
Product for vaginal use	**115 (36%)**	**71 (23%)**	**1.89 (1.33–2.68)**	**1.68 (1.16–2.43)**
Product for rectal use	16 (5%)	11 (4%)	1.43 (0.65–3.13)	

Bold values indicate significant findings

Education, parity, primary residence and prior vaginal product use were not associated with interest in a daily pill (Wald chi-square p > 0.05)

^a^Not answered includes missing and prefer not to answer

^b^May add up to more than 100% as more than one response was possible

#### cMPT

As noted above, 82% of respondents (n = 518) would be more likely to use a cMPT than a product for HIV/STI prevention only. As shown in Table 4, in the multivariate model, predictors of preference for a cMPT were wanting products that could be applied vaginally (AOR 2.15, 95% CI 1.19–3.89), prevent HIV (AOR 1.70, 95% CI 1.06–2.70), or prevent pregnancy (AOR 1.81, 95% CI 1.16–2.82) when asked about each separately.

#### On-Demand Methods

As shown in Table 5, 551 women (87%) were interested in using at least one of the five on-demand methods (vaginal gel inserted before or after sex, vaginal film or FDI inserted before sex, oral pill taken before sex). On-demand product interest was associated with previous use of female condoms (AOR 12.56, 95% CI 1.65–95.64), spermicides and/or diaphragm (AOR 7.74, 95% CI 1.37–43.72), or injectables (AOR 1.90, 95% CI 1.04–3.50); desiring products that could be applied vaginally (AOR 2.63, 95% CI 1.20–5.76); and interest in products to prevent HIV (AOR 2.09, 95% CI 1.18–3.70) or cMPTs (AOR 2.07, 95% CI 1.18–3.65).

By contrast, prior IVR use was associated with significantly lower interest in on-demand methods (AOR 0.29; 95% CI 0.11–0.78). In addition, women from Latin America were four times as likely to find on-demand methods appealing compared to those from sub-Saharan Africa (AOR 4.08; 95% CI 1.17–14.19). In the bivariate model, women who had multiple sexual partners were nearly three times more likely to be interested in using on-demand methods than women with one partner or no partner (OR 2.88; 95% CI 1.01–8.12), however, the association was not present in the multivariate model which adjusted for primary residence, contraceptive methods used, prevention needs, interest in cMPT and formulation preference.

#### Long-Acting Methods

As shown in Table 6, 490 women (78%) would use at least one of the long-acting methods (injectable, IVR, implant). Prior use of contraceptive implants (AOR 2.43, 95% CI 1.17–5.02) or injectables (AOR 2.18, 95% CI 1.27–3.74), or desire for a cMPT (AOR 3.14, 95% CI 1.96–5.04) or an HIV prevention product (AOR 1.98, 95% CI 1.27–3.07) were associated with higher odds of wanting to use a long-acting method. In addition, women with more than 12 years of education had significantly lower odds of interest in long-acting methods than those with 1–12 years of education (AOR 0.46; 95% CI 0.28–0.77).

#### Daily Pill

About half of the respondents (n = 320) were interested in the daily pill for HIV/STI prevention (Table 7). Women who had used female condoms for HIV/STI prevention (AOR 2.15, 95% CI 1.32–3.50) and those who wanted an HIV prevention method (AOR 1.88, 95% CI 1.26–2.80) or a product that could be applied vaginally (AOR 1.68, 95% CI 1.16–2.43) had higher odds of interest in a daily pill, whereas those who had ever used an IUD were significantly less likely to be interested in a daily pill (AOR 0.59, 95% CI 0.41–0.86).

## Discussion

Share.Learn.Shape was the first global online survey to explore women’s opinions about HIV/STI prevention products in development. Most respondents were interested in using a variety of products, with few interested in using only on-demand or only long-acting formulations. The variability in desirable product attributes highlights the imperative to support ongoing development of different prevention technologies. Regardless of their varying preferences for product types and formulations, women emphasized safety, affordability, accessibility, efficacy, ease of use, and lack of impact on sex as the most important characteristics of any HIV/STI prevention product.

Although exploring the reasons for cMPT preference was beyond the scope of our survey, other studies have suggested that key reasons might include discretion, convenience, and reduced stigma compared to use of methods solely for HIV/STI prevention [35, 36]. Over 80% of participants said they would be more likely to use a cMPT than a product for HIV/STI prevention only, with similar interest across geographic and demographic groups. Our findings align with previous studies indicating that most women would prefer HIV/STI prevention methods that also contain a contraceptive [9, 10, 12, 26, 31, 35, 37, 38].

We found that desiring a product for HIV prevention was consistently a significant predictor of interest in all three product types (on-demand, long-acting or daily pill), and in cMPTs. Most women who were interested in using cMPTs were interested in both on-demand and long-acting formulations, although prior experience with a type of method was often associated with increased interest in a similar type of method. For example, those who used vaginal on-demand methods in the past, such as female condoms, were significantly more likely to be interested in on-demand methods, whereas women who had used implants were significantly more likely to be interested in long-acting methods. An unexpected finding was that women who had used injectable contraception expressed interest in both on-demand and long-acting methods, highlighting the differing needs among this sub-group. Prior injectable users may have had variable experiences with use, leading them to be open to both long-acting and on-demand methods.

Another interesting finding was that, for the most part, we did not find any significant associations with product interest and age group, marital status, education, or region, with the exception that women from Latin America were more interested in on-demand products than those from sub-Saharan Africa. Our findings support previous research about women’s complex decision-making processes when selecting contraception and HIV/STI prevention that includes individual preferences, accessibility of methods, partner considerations and shifting priorities over their lives [38–44]. As has been found in other studies, for some women safety or lack of side effects was of paramount concern, whereas for others, HIV prevention efficacy was prioritized over all other product characteristics [36].

### Limitations

Our study had several limitations. First, the respondents answered questions about interest using hypothetical products in development and did not use actual products. As has previously been articulated, hypothetical research is limited, with uptake likely to be lower or higher once any product becomes available [45–47]. Second, we did not ask any direct questions about perception of risk for HIV, STIs, or pregnancy. However, we did ask which types of products women were most interested in using. Given that a product to prevent HIV was cited by the majority of women (76%), it is likely that most respondents did perceive themselves to be at some risk of HIV and were appropriate participants in the survey. In addition, our sample population was 30 years old, on average, which is older than those at greatest risk of HIV. However, approximately 25% of the population was 18–24 years old, which provides important data for young women, who are at highest risk of HIV acquisition. A third limitation is the lack of generalizability of our data, given the recruitment of a convenience sample. The final analysis included 630 self-selected participants, who had to have access to a cell phone or computer and the internet and had to be sufficiently literate to self-administer the survey. As such, our respondents were likely to represent a higher-than-average socioeconomic status and were more likely to come from urban or peri-urban areas than rural settings. However, as younger, more “tech” savvy individuals are expected to be early adopters of any new product, we believe the information gathered from this study still provides an excellent starting point for continued development of MPTs and other HIV/STI prevention technologies. And, although it is impossible to confirm that the participants were unique individuals, given the lack of compensation for participating, there was no incentive to feign eligibility or take the survey more than once. Another limitation is our difficulty recruiting respondents from Europe and Asia, which limits the utility of our findings for those regions. Missing data also limited our ability to interpret the findings. Since approximately 10% of respondents did not indicate which country they were from, we were unable to fully describe the population and could not conduct analyses by region. We also do not know why respondents did not answer specific questions—whether it was due to lack of interest in a specific product, survey fatigue, or inadvertently skipping a question. Finally, we do not know if respondents viewed the video clips.

## Conclusions

Our results reinforce previous studies that have found that most women would be more likely to use a cMPT versus a product solely for HIV/STI prevention, regardless of demographics or geography. Although no single product will be preferred by all users under every circumstance, combining HIV/STI prevention with contraception is likely to make any product more acceptable to most women. Given the broad interest and potential for cMPTs to have a significant impact on improving women’s sexual and reproductive health, future product development efforts should focus on an array of cMPTs to expand women’s HIV/STI and prevention options.

## Data Availability

Available upon request.

## Code Availability

Available upon request.
